# Multi-mode of Four and Six Wave Parametric Amplified Process

**DOI:** 10.1038/srep43689

**Published:** 2017-03-03

**Authors:** Dayu Zhu, Yiheng Yang, Da Zhang, Ruizhou Liu, Danmeng Ma, Changbiao Li, Yanpeng Zhang

**Affiliations:** 1 Key Laboratory for Physical Electronics and Devices of the Ministry of Education & Shaanxi Key Lab of Information Photonic Technique, Xi’an Jiaotong University, Xi’an 710049, China

## Abstract

Multiple quantum modes in correlated fields are essential for future quantum information processing and quantum computing. Here we report the generation of multi-mode phenomenon through parametric amplified four- and six-wave mixing processes in a rubidium atomic ensemble. The multi-mode properties in both frequency and spatial domains are studied. On one hand, the multi-mode behavior is dominantly controlled by the intensity of external dressing effect, or nonlinear phase shift through internal dressing effect, in frequency domain; on the other hand, the multi-mode behavior is visually demonstrated from the images of the biphoton fields directly, in spatial domain. Besides, the correlation of the two output fields is also demonstrated in both domains. Our approach supports efficient applications for scalable quantum correlated imaging.

Multi-mode is a fundamental characteristic for quantum information technologies^1^,^2^. The capacity of a communication system will be extremely expanded with multi-mode in the frequency domain, while quantum imaging is possible only when different spatial regions belong to different modes^3^,^4^. Recently, multi-mode properties shared between correlated^5^,^6^ and entangled fields^7^,^8^,^9^,^10^ have attracted immense attention. One of the competitive candidates is a pair of correlated fields^11^ by non-degenerate four-wave mixing (FWM) process in rubidium vapor, where hundreds of spatial modes could be achieved^12^. The FWM system has no requirement for an optical cavity due to the embedded nonlinearity and spatial separation of the twin output fields^13^, so it is easy to be adjusted in the experiment. Moreover, the FWM system has narrow bandwidth and high generation rate^14^ and could be treated as a phase-insensitive amplifier^15^ with low noise. Thus it could be applied to further multi-mode configurations such as cascaded FWM^16^ process and quantum network, while it is also favorable in quantum entangled imaging^17^,^18^,^19^, nonclassical squeezing states^20^,^21^,^22^ and coherent slow light.

In this paper, we deepen the multi-mode research in a system coexisting of four- and six-wave mixing (SWM)^23^,^24^. Compared with the pioneering FWM configuration^25^, our system shows better responses to the dressing modulation. In frequency domain, the multi-mode appears as one non-degenerate signal peak splits into multiple peaks on the spectrum. Such splitting can be attributed to the both the internal and external dressing effects^26^. Then in the spatial domain, we observed that different points on the light-spot image show non-synchronous responses versus the change of detuning. This provides a solid evidence that those points are in different spatial mode, from which we can deduct one hundred or more spatial modes exist in one beam. Besides, the intensity correlation can be observed in both domains^27^.

## Results

**Figure 1:**
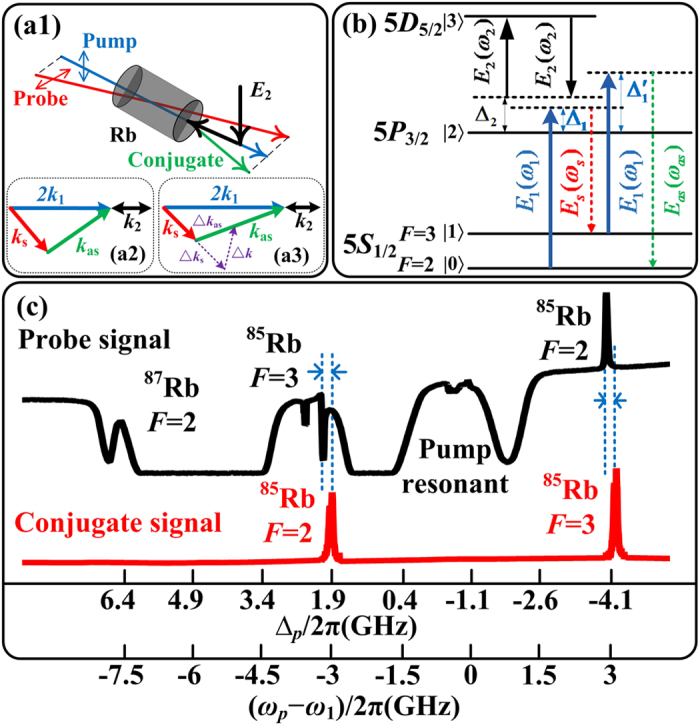
(**a1**) Spatial beams alignment of the PA-SWM process. (**a2**) Phase-matching geometrical diagram of the PA-SWM process. (**a3**) Phase mismatching geometrical diagram. (**b**) Energy-level diagram for the inverted-Y configuration in ^85^Rb vapor. (**c**) Measured probe transmission signal and corresponding conjugate signal versus the probe detuning.

At the beginning, when we only apply a strong pumping field ***E***_1_, the three level “double-Λ” rubidium (Rb) atomic configuration is formed, involving two hyperfine ground states of 5S_1/2_ [*F* = 2 (|0〉) and *F* = 3 (|1〉)] and an excited state 5P_3/2_ (|2〉). The spatial beams alignment and energy-level diagram are shown in Fig. 1(a1,b), respectively. When ***E***_1_ (frequency *ω*_1_, wave vector ***k***_1_, Rabi frequency *G*_1_, vertical polarization) is set as 780.23 nm with power up to 100 mW and the temperature of media set as around 145 °C, a spontaneous parametric^4^ FWM process is triggered and generates a pair of Stokes (*ω*_*s*_) and anti-Stokes (*ω*_*as*_) fields. The two generated fields are symmetric to the axis of ***E***_1_ with the angle of 0.26°, satisfying the phase matching conditions ***k***_*s*_ = 2***k***_1_ − ***k***_*as*_ and ***k***_*as*_ = 2***k***_1_ − ***k***_*s*_. Then the weak probe field ***E***_*p*_ (*ω*_*p*_, ***k***_*p*_, *G*_*p*_, horizontal polarization, 400 *μ*W) intersects with ***E***_1_ inside the Rb cell with the same angle of 0.26°. When the frequency of ***E***_*p*_ is the same as Stokes or anti-Stokes signal (*ω*_*p*_ = *ω*_*s*_ or *ω*_*p*_ = *ω*_*as*_), ***E***_*p*_ could be treated as injected into Stokes or anti-Stokes field, while the other non-injected port is termed as the conjugate channel. Thus the parametric amplified^28^ four-wave mixing (PA-FWM) is formed by the injection, where both Stokes and anti-Stokes fields are amplified. The intensities of Stokes and anti-Stokes fields are proportional to 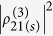 and 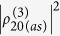, respectively. Here 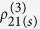 and 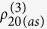 are the third-order density matrix elements, obtained by the Liouville pathways (perturbation chains) 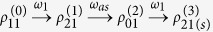
(Stokes field) and 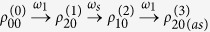 (anti-Stokes field), and shown as


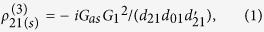



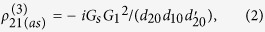


where *G*_*ij*_ = *μ*_*ij*_*E*_*ij*_/*ħ (j* = *s* and *as*) is the Rabi frequency, Γ_*ij*_ = (Γ_*i*_ + Γ_*j*_)/2 is the decoherence rate between |*i*〉 and |*j*〉; Δ_*i*_ is the detuning between the resonant transition frequency Ω_*i*_ and the laser frequency *ω*_*i*_ of ***E***_*i*_, denoted as ∆_*i*_ = Ω_*i*_ − *ω*_*i*_. And we define 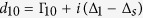, 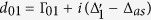, 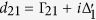, 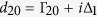, 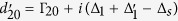, 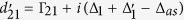. 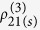 causes the nonlinear gain peak in Stokes field at the non-degenerate window Δ_1_ − Δ_*s *_= 0 when probe beam has the same frequency with Stokes field, i.e. Δ_*p*_ = Δ_*s*_, where 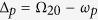 is the detuning of probe field. While 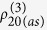 causes nonlinear gain in anti-Stokes field at 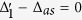 when Δ_*P*_ = Δ_*as*_.

Next, we add ***E***_2_ (*ω*_2_, ***k***_2_, *G*_2_, wavelength of 776 nm) at the opposite direction of ***E***_1_ as injection into Stokes field, shown in Fig. 1(a1,b). ***E***_2_ acts as a dressing field connecting the transition 5P_3/2_ (|2〉) to 5D_5/2_ (|3〉). The presence of ***E***_2_ will lead to an electromagnetically induced absorption (EIA)^29^, i.e. a dip in the probe transmission signal. If the EIA overlaps with the PA-FWM gain peak, the parametric amplified six-wave mixing (PA-SWM) is formed in the four-level inverted Y-type atomic configuration. The phase matching conditions for the PA-SWM are ***k***_*s*_ = 2***k***_1_ − ***k***_*as*_ + ***k***_2_ − ***k***_2_ and ***k***_*as*_ = 2***k***_1_ − ***k***_*s*_ − ***k***_2_ + ***k***_2_, shown in Fig. 1(a2). Then the intensity of PA-SWM is associated with the fifth-order density matrix elements, 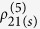 and 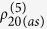, which are introduced by the perturbation chain 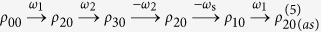 (anti-Stokes field) and 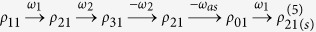 (Stokes field). Thus, the overall multi-wave mixing (MWM)^30^ system, i.e. the co-existing of PA-FWM and PA-SWM could be shown as FWM signal dressed by ***E***_2_. Then Eqs (1 and 2) can be modified as


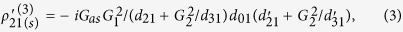



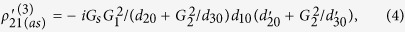


where the terms are defined as following: 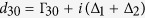, 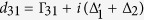, 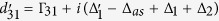, 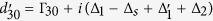. One can easily prove that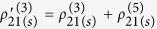 and 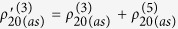 through Taylor Expansion.

Experimentally, we scan the probe detuning ∆_*p*_ over 14.0 × 2π GHz when ∆_1_ is around −1.1 × 2π GHz, then the signals in both probe and conjugate channels are shown on spectrum as Fig. 1(c). There are two positions triggering the non-degenerate MWM signals of ^85^Rb: ∆_*p*_ = 1.9 × 2π GHz and −4.1 × 2π GHz, which correspond to *ω*_*p*_−*ω*_1_ = −3.0 × 2π GHz and 3.0 × 2π GHz, respectively. Here *ω*_*p*_ − *ω*_1_ < 0 means probe beam is injected into Stokes field, while *ω*_*p*_−*ω*_1_ > 0 shows it is injected into anti-Stokes field; *ω*_*p*_−*ω*_1_ = 0 GHz is the resonance point. Specifically, at *ω*_*p*_−*ω*_1_ = −3.0 × 2π GHz, the transition of probe field is *F* = 3 → *F*′, and the conjugate is *F* = 2 → *F*′; on the contrary, at *ω*_*p*_−*ω*_1_ = 3.0 × 2π GHz, the probe and conjugate fields stem from ^85^Rb, *F* = 2 → *F*′ and ^85^Rb, *F* = 3 → *F*′ transitions, respectively. The two triggering points are symmetric to *ω*_*p*_ − *ω*_1_ = 0, and the 6 × 2π GHz frequency difference between the two points is exactly the frequency gap between Stokes and anti-Stokes fields. The strong symmetry suggests the Stokes and anti-Stokes fields are strongly correlated both in frequency and spatial domains.

Generally, the probe and conjugate non-degenerate peaks raise exactly at the same ∆_*p*_ in each case, while sometimes they have a frequency deviation (denoted in Fig. 1(c)), which stems from the relaxed phase matching condition^12^. As Stokes and anti-Stokes fields are beams with certain bandwidths, we can define *ϖ*_*s*_, *ϖ*_*as*_ as the central frequencies of Stokes and anti-Stokes signals, respectively, in the ideal phase matching condition. Then the realistic frequencies are obtained as *ω*_*s*_ = *ϖ*_*s*_ + *δ* and *ω*_*as*_ = *ϖ*_*as*_ − *δ*, where *δ* is the phase-mismatch in frequency.

In addition, we introduce vector ∆***k*** denoting the phase mismatch in space^31^,^32^, where


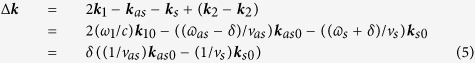


The equation shows the relationship between ∆***k*** and *δ*, and the phase mismatching diagram is shown in Fig. 1(a3). *vs, vas* are the group velocities of Stokes and anti-Stokes signals, and ***k***10, ***k**s*0, ***k**as*0 are the unit vectors of pump, Stokes and anti-Stokes beams, respectively. Since phase mismatch indicates the multi-mode properties of signals, the relationship of *δ* and ∆***k*** demonstrates the concord of multi-mode in both frequency and spatial domains. The detailed deductions and expressions of theoretical models are in Supplementary Information.

**Figure 2:**
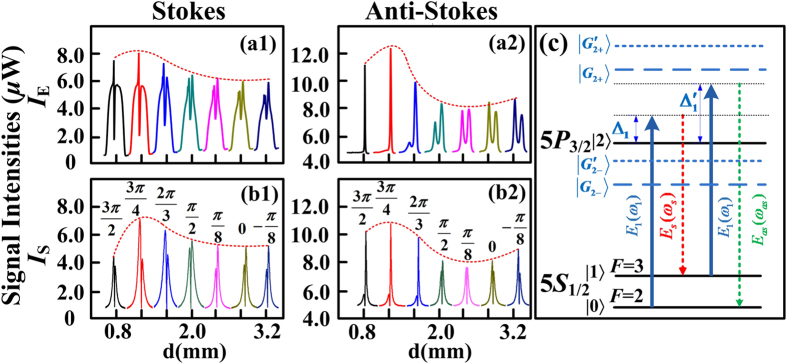
Intensity evolutions of Stokes (**a1**) and anti-Stokes (**a2**) PA-FWM signals versus ∆_*p*_ in probe field by increasing the diameter of pump beam ***E***_1_. (**b1,b2**) Normalized simulations corresponding to (**a1,a2**), respectively. (**c**) Dressing energy diagram of PA-FWM.

We begin with the case of frequency multi-mode through FWM. Here only pump beam ***E***_1_ and probe beam ***E***_p_ interact inside the rubidium vapor, which forms a PA-FWM process. Due to the strong internal dressing effect of ***E***_1_, the Stokes or anti-Stokes peak splits into two peaks in Fig. 2(a1,a2). That is the so-called Autler-Townes splitting^33^, where the excited state 5P_3/2_ (|2〉) is splited by ***E***_1_. Multiple split-peaks are direct evidences of multi-mode in frequency domain. Specifically, by increasing the diameter of ***E***_1_, the intensities of both Stokes and anti-Stokes PA-FWM gain peaks first grow to maxima then decrease. Moreover, there exists the competition between the left and right portion of the signal: in Fig. 2(a1) of Stokes signal, the left peak dominates at the beginning, then the difference of the left and right peak vanishes gradually, finally the right peak becomes more prominent; while the case of anti-Stokes in Fig. 2(a2) is just the contrary.

Here the splitting is caused by the internal-dressing effect of ***E***_1_, whose diameter is associated with the nonlinear phase modulation^34^. Since the intensities of Stokes and anti-Stokes gains are proportional to the square form of density matrix elements 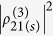 and 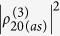 respectively, where


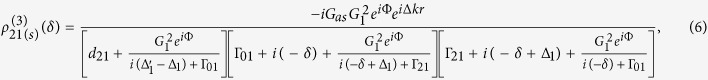



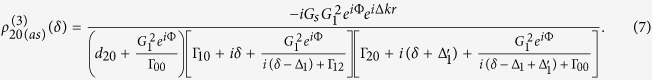


For the sake of frequency multi-mode, the phase mismatch term *δ* is introduced. Besides, an additional nonlinear phase shift factor *e*^*i*Φ^ is introduced^35^ via cross-Kerr effect^36^ from the internal-dressing effect of ***E***_1_, compared with Eqs (1 and 2). See Methods section for the theory of nonlinear phase shift and cross-Kerr effect. The maxima of 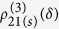 and 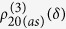 occur at 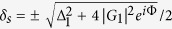 and 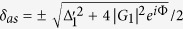, respectively, which further correspond to the enhancement conditions 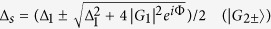 and 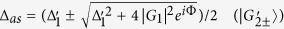. While *δ*_*s*_ = 0 and *δ*_*as*_ = 0 correspond the suppression conditions Δ_*s*_ = Δ_1_ and 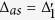, respectively. The parameters are Δ_1_ = −1.1 × 2*π* GHz, 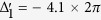 GHz and the detailed dressing energy diagram is showed in Fig. 2(c). Thus, the splitting of single peak into double is caused by enhancement conditions, while the dip between the double peaks corresponds to the suppression condition. By adjusting the pump beam diameter, the relative position of pump and probe beams will be changed, which tends to vary the nonlinear phase shift Ф. Theoretically, the change of Ф leads to the movement of ***E***_1_-induced splitting energy levels, which means the switch of enhancement and suppression. And when the diameter changes from 0.8 mm to 3.2 mm, Ф goes through 1.625 π. The normalized theoretical simulations (Fig. 2(b1,b2)) fit perfectly with experiments (Fig. 2(a1,a2)). Besides, varying Ф also changes the distance of splited peaks from 20 to 300 MHz, which is the bandwidth of *δ*, i.e. the bandwidth of multimode. Concisely, multi-mode could be caused by internal dressing effect, and the properties are controlled by nonlinear phase shift.

**Figure 3:**
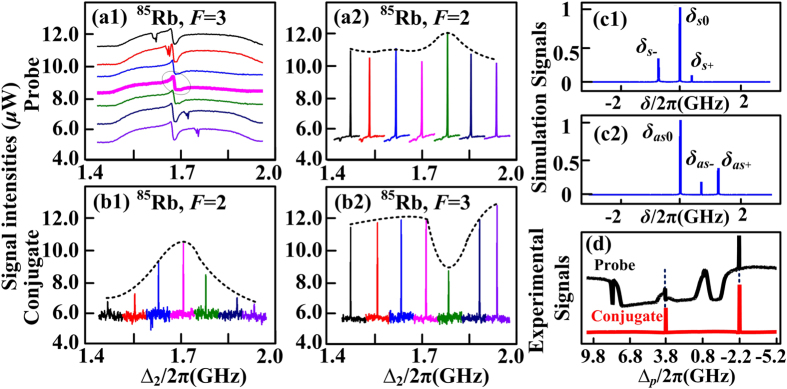
Measured probe and corresponding conjugate signals versus ∆_*p*_ at different dressing detuning ∆_2_. The wavelength of pump beam ***E***_1_ is 780.2356 nm (∆_1_ = −1.07 × 2π GHz). Intensity evolutions of Stokes (**a1**) (*ω*_*p*_ − *ω*_1_ = −3 × 2π GHz) and anti-Stokes (**a2**) (*ω*_*p*_ − *ω*_1_ = 3 × 2π GHz) signals of the PA-SWM & FWM in probe field versus ∆_*p*_ by increasing ∆_2_. (**b1,b2**) Signals in conjugate channel corresponding to (**a1,a2**). (**c1,c2**) Simulations of Stokes and anti-Stokes signals versus frequency mismatch *δ*, respectively. (**d**) Overview probe and corresponding conjugate spectrograms versus ∆_*p*_ over 14 × 2π GHz.

Next, we focus on the properties of frequency multi-mode in SWM process by adding a dressing beam ***E***_2_. In order to avoid the internal-dressing effect, power of ***E***_1_ is lowered. In Fig. 3(a1), it is observable that ***E***_2_ will lead to a small dip on the intensity profile of PA-FWM, which is caused by EIA of ***E***_2_. Increasing ∆_2_ over 0.3 × 2π GHz around 776.1605 nm (∆_2_ = 1.65 × 2π GHz), the EIA dip moves with ∆_2_ in Fig. 3(a1); when the EIA overlaps exactly with the PA-FWM gain peak (circled in Fig. 3(a1)), the PA-SWM process will be formed and the anti-Stokes gain peak (Fig. 3(a2)) will be enhanced in probe channel, and the corresponding anti-Stokes signal in conjugate channel (Fig. 3(b1)) is enhanced as well. However, due to the phase-matching condition ***k***_*s*_ = 2***k***_1_ − ***k***_*as*_ + ***k***_2_ − ***k***_2_ and energy conservation in Stokes and anti-Stokes fields, the enhancement of anti-Stokes signal leads to the Stokes peak in conjugate channel (Fig. 3(b2)) impaired.

Theoretically, considering the phase mismatch caused by the external dressing effect of ***E***_2_, Eqs (3 and 4) can be expressed as


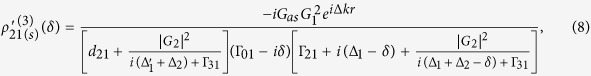



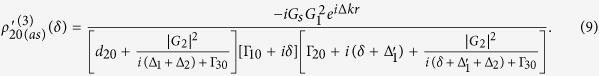


Here with the parameters set as ∆_1_ = −1.07 × 2π GHz and ∆_2_ = 1.65 × 2π GHz and power of ***E***_2_ being 24 mW, the normalized simulations of Eqs (8 and 9) are shown as Fig. 3(c1,c2) for Stokes and anti-Stokes signals, respectively. Both Stokes and anti-Stokes signals have three maxima, denoted as *δ*_*s*−_, *δ*_*s*0_, *δ*_*s*+_, and *δ*_*as*−_, *δ*_*as*0_, *δ*_*as*+_, where *δ*_*s*0_ = *δ*_*as*0_ = 0. The intensities at *δ*_*s*0_ = *δ*_*as*0_ = 0 are much greater than others, so this case happens only at *δ*_*s*0_ = *δ*_*as*0_ = 0, which means no frequency mismatch. To sum up, weak external dressing effect will not lead to multi-mode.

**Figure 4:**
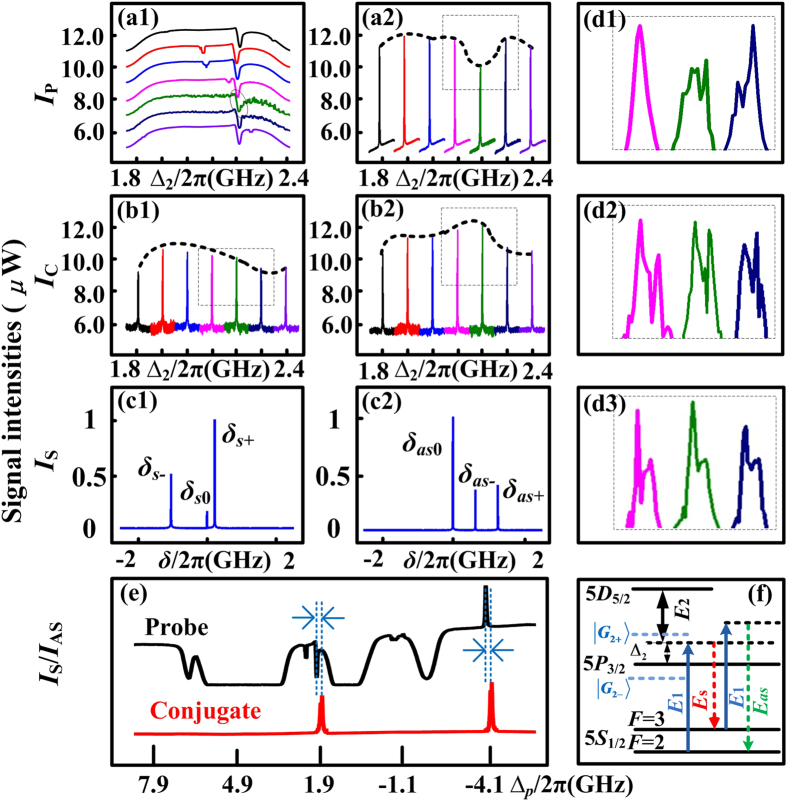
Measured probe and corresponding conjugate signals versus ∆_*p*_ at different dressing detuning ∆_2_. with detuning of pump beam ***E***_1_ set as ∆_1_ = −1.61 × 2π GHz, the intensity evolutions of Stokes (**a1**) (*ω*_*p*_−*ω*_1_ = −3 × 2π GHz) and anti-Stokes (**a2**) (*ω*_*p*_−*ω*_1_ = 3 × 2π GHz) PA-SWM & FWM signals in probe channel versus ∆_*p*_ by increasing ∆_2_. (**b1,b2**) Signals in conjugate channel corresponding to (**a1,a2**), respectively. (**c1,c2**) Normalized simulations of Stokes and anti-Stokes signals versus *δ*, respectively. (**d1,d2,d3**) Detailed splitting of (**a2,b1,b2**), respectively. (**e**) Overview probe and conjugate spectrograms versus ∆_*p*_ over 14 × 2π GHz. (**f**) Dressing energy level diagram.

Subsequently, in Fig. 4 we discuss the case that SWM multi-mode formed by strong external dressing effect of ***E***_2_. Here, the detuning of pump field ***E***_1_ is ∆_1_ = −1.61 × 2π GHz, ∆_2_ = 2.15 × 2π GHz, and power of ***E***_2_ is 80 mW, i.e. with larger detunings and greater dressing power. Hence the dressing effect is more obvious, and the dressing energy diagram is shown in Fig. 4(f). Experimentally, when the ***E***_2_-induced EIA overlaps with PA-FWM signal (circled on Fig. 4(a1)), the PA-SWM is generated. However, the anti-Stokes signal in probe field splits further into three or more smaller peaks, highlighted in Fig. 4(d1). The splitting will cause the peak intensity to lower down, as well as anti-Stokes signal in conjugate field to be impaired (Fig. 4(b1)), whose splitting details are shown in Fig. 4(d2). Due to the energy conservation in Stokes and anti-Stokes fields, the suppression in anti-Stokes field leads to the enhancement of Stokes field (Fig. 4(b2)), while Stokes signal also splits into multiple peaks (Fig. 4(d3)).

In contrast with Fig. 2, external dressing effect of SWM causes division to at least three peaks, while internal dressing effect of FWM only causes double peaks. This suggests SWM will lead to more modes than FWM system. Besides, the Stokes and anti-Stokes signals will not appear simultaneously at the same ∆p as shown in Fig. 3(e), where the deviation is the phase mismatch *δ* in frequency domain. We can measure that *δ* = 186.66 MHz here.

Theoretically, we also conduct Stokes and anti-Stokes simulations versus phase mismatch *δ* by Eqs (8 and 9), shown in Fig. 4(c1,c2). There are still three maxima for every signal. However, compared with Fig. 3(c1,c2), the relative intensities at nonzero *δ*_*s*−_(*δ*_*as*−_), *δ*_*s*+_(*δ*_*as*+_) to *δ*_*s*0_(*δ*_*as*0_) are stronger, which means the Stokes (anti-Stokes) signal has larger probability to appear at *δ*_*s*−_(*δ*_*as*−_) or *δ*_*s*+_(*δ*_*as*+_). The simulations suggest that stronger dressing effect will result in larger probability of phase mismatch and multi-mode. In short, the multi-mode phenomenon of FWM is caused by internal dressing effect, while in case of SWM, it is subjected to strong dressing effect of ***E***_2_. Besides, SWM has better potential to achieve more modes than FWM.

**Figure 5:**
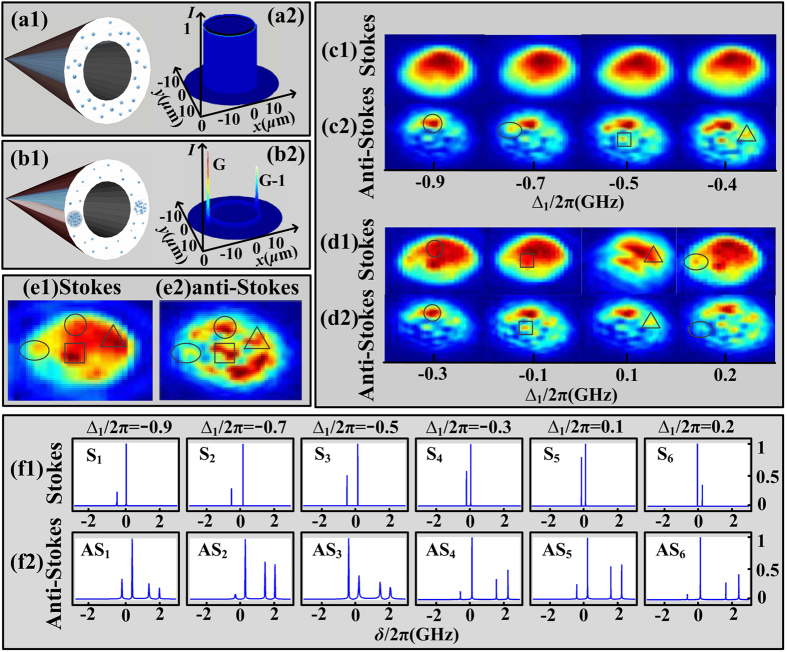
Light spot images of Stokes and anti-Stokes fields. The emission cone (**a1**) and intensity distribution (**a2**) without probe beam ***E***_*p*_ injected. (**b1,b2**) The emission cone and intensity distribution with probe ***E***_*p*_ turned on. (**c1,c2**) The spatial images of Stokes (upper row) and anti-Stokes field (lower row) via increasing ∆_1_ from −0.9 × 2π to −0.4 × 2π GHz. (**d1,d2**) The same as (**c1,c2**) but ∆_1_ from −0.3 × 2π to 0.2 × 2π GHz, respectively. (**e1,e2**) The detailed light spot images of Stokes and anti-Stokes fields at ∆_1_ = 0.3 × 2π GHz, respectively. (**f1,f2**) Normalized simulations of Stokes (upper row) and anti-Stokes (lower row) signals versus *δ* at different ∆_1_.

After discussion about multi-mode in frequency domain, in Fig. 5 we study multi-mode in spatial domain through light-spot images (detected by a charge coupled device camera, CCD) of probe and conjugate fields. We set ∆_*p*_ = ∆_s_ so the probe field is injected into Stokes field (while the conjugate channel means anti-Stokes field), and change Δ_1_ from −0.9 × 2π GHz to 0.3 × 2π GHz. Theoretically, with the phase-mismatch *δ* in frequency, we have ∆***k*** = *δ*((1/*v*_*as*_)***k***_*as*0_ −  (1/*v*_*s*_)***k***_*s*0_) from Eq. (5), then spatial multi-mode appears. Provided ***E***_*p*_ is not injected, each pair of correlated Stokes and anti-Stokes photons could distribute evenly between the ideal Stokes and anti-Stokes cones due to the perfect symmetry^37^ (Fig. 5(a1)), so at a certain cross-section of pump axis, the intensity of photon appears evenly on a circular ring (Fig. 5(a2)). Next, when applying ***E***_*p*_ in our experiments, the intensities in probe and conjugate fields are amplified with multiples of *G* and (*G*−1)^38^ (Fig. 5(b1,b2)), which provides a better chance for the detection, but the spatial multi-mode distribution is unaffected.

Experimentally, the detailed spatial images of both probe (Stokes) and conjugate (anti-Stokes) fields are shown. Initially, we focus on the evolutions of Stokes and anti-Stokes spatial images versus ∆_1_ in Fig. 5(c1. Generally, the size of both fields become larger by increasing ∆_1_; and the fields intensities are strengthened. Specifically, the shape of the spatial mode varies with ∆_1_. In Fig. 5(c1), the Stokes image remains almost the same as the intensity is too strong that we cannot observe too many details. While in Fig. 5(c2) for anti-Stokes, at first (Δ_1_ = −0.9 × 2π GHz) there is only one small bright spot (denoted on figures by circle), later three more spots (denoted on figures by oval, square and triangle, respectively) appear subsequently as Δ_1_ gradually changes to −0.4 × 2π GHz. As the four spots have different responses for ∆_1_, it testimonies that the four spots represent different spatial modes. So instead of using noise figure (NF)^12^, we prove spatial multi-mode via carefully analyzing the details of light spot images. Moreover, the intensity correlation is also proved. For example, with changing Δ_1_ in Fig. 5(d1,d2), one pair of corresponding points of Stokes and anti-Stokes fields (denoted by circles) are getting bright or dark together from −0.3 × 2π to 0.2 × 2π GHz, which means they are correlated in intensity. Other pair of points denoted by squares, triangles and ovals are also correlated, respectively. This phenomenon directly suggests the corresponding spatial modes in Stokes and anti-Stokes fields are strongly correlated in intensity, while the study on noise and squeezing correlation has been presented by Paul Lett group previously^4^,^11^.

The observations can be clearly interpreted by the theory. Applying Eqs (6 and 7) to show the dressing effect of ***E***_1_, the normalized simulations of Stokes and anti-Stokes signals versus *δ* at different ∆_1_ are shown as Fig. 5(f1,f2). The Stokes simulations have two discrete peaks while anti-Stokes have four peaks, and the positions of the peaks are still denoted by *δ*_*s*_ and *δ*_*as*_, respectively. Firstly, the absolute values of *δ*_*as*_ are generally much larger than *δ*_*s*_, so it is reasonable that there are more spatial modes in anti-Stokes than Stokes field. The deduction fits well with the appearance that there are more light spots in the images of anti-Stokes (Fig. 5(c2,d2)) than Stokes (Fig. 5(c1,d1)). Moreover, by changing ∆_1_, the absolute value of *δ*_*as*_ generally grow higher (Fig. 5(f2), from left to right), which indicates larger phase mismatch and more spatial multi-modes. It is true that we have more modes with changing ∆_1_, in Fig. 5 (c2,d2, from left to right).

The detailed spot images and multi-mode correlation at ∆_1_ = 0.3 GHz are shown in Fig. 5(e1,e2). The number of modes is calculated as *N* = *A*_(*QPM*)_/*A*_(*one*)_, where *A*_(*QPM*)_ is the quasi-phase-matched area, *A*_(*one*)_ is the area of one mode^3^. If we assign the circled mode as a standard mode area, by comparing the area of the single mode and the whole circular light spot image, it can be calculated that we have totally 112 spatial modes in our observations. So at least one hundred spatial modes exist in one field, and the spatial modes of Stokes and anti-Stokes fields are correlated.

## Methods

### Experimental setup

The pump beam ***E***_1_ is emitted from a continuous Ti:sapphire laser of 20–100 mW, meanwhile the probe beam ***E***_*p*_ is excited by an external cavity diode laser (ECDL). The two beams are of orthogonal polarization, intersecting at the rubidium cell, which couple the three-level “double-Λ” PA-FWM configuration. If dressing beam ***E***_2_ (30 mW, from another ECDL) is injected opposite to ***E***_1_, the four-level “inverted-Y” PA-SWM process is formed. The 1 cm rubidium cell is of natural abundance, including both ^85^Rb and ^87^Rb, and wrapped by *μ*-metal and heated by a heater tape. Both the probe and conjugate signals are detected by a balanced photodetector (10^5^ V/A) with the diameter of around 0.5 cm, or shot by a CCD.

### Nonlinear phase shift and cross-Kerr effect

The so-called cross-Kerr effect occurs when a weak probe beam interacts with a strong pump beam, then the spatial orientation of probe beam will be shifted, and a nonlinear term, *n*_2_, will be added into the refractive index at the intersection point. Here we have 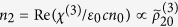 where the density matrix element 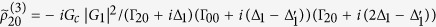, *G*_c_ is the Rabi frequency of conjugate field, and *χ*^(3)^ is the third-order nonlinear susceptibilities. The nonlinear phase shift Ф is introduced as 
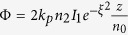
, where *k*_*p*_ is the length of probe field wave vector.

## Additional Information

**How to cite this article:** Zhu, D. *et al*. Multi-mode of Four and Six Wave Parametric Amplified Process. *Sci. Rep.* **7**, 43689; doi: 10.1038/srep43689 (2017).

**Publisher's note:** Springer Nature remains neutral with regard to jurisdictional claims in published maps and institutional affiliations.

## Supplementary Material

Supplementary Information
