# Supplementary material for: Multi-mode of Four and Six Wave Parametric Amplified Process
Source: Sci Rep. 2017 Mar 3;7:43689. doi: 10.1038/srep43689 (PMC5334647; doi:10.1038/srep43689)
Supplement: Supplementary Information [file srep43689-s1.pdf]

Supplementary Information for "Multi-mode of Four and Six Wave Parametric Amplified Process"

Dayu Zhu, Yiheng Yang, Da Zhang, Ruizhou Liu, Danmeng Ma, Changbiao Li and Yanpeng Zhang*

Key Laboratory for Physical Electronics and Devices of the Ministry of Education & Shaanxi Key Lab of Information Photonic Technique, Xi'an Jiaotong University, Xi'an 710049, China

*Corresponding authors: ypzhang@mail.xjtu.edu.cn

The Supplementary Information mainly provides the theoretical derivations and equations of the quantum output of Stokes and anti-Stokes fields, the characteristics of signals consisting of nonlinear gain and electromagnetically induced absorption (EIA), and the introduction of wave packet function.

To begin with the simplest case, when we only apply a strong pumping field $\boldsymbol{E}_1$, blocking probe field $\boldsymbol{E}_p$ and dressing field $\boldsymbol{E}_2$, the three level "double-Λ" rubidium (Rb) atomic configuration is formed, involving two hyperfine ground states of $5S_{1/2}$ [F=2 ($|0\rangle$) and F =3($|1\rangle$)] and an excited state $5P_{3/2}$ ($|2\rangle$). The spatial beams alignment and energy-level diagram are shown in, respectively. With $\boldsymbol{E}_1$ of 780.23 nm and up to 100 mW (frequency ω_1, wave vector $\boldsymbol{k}_1$, Rabi frequency G_1, vertical polarization) at around 145 °C, the SP-FWM is triggered and generates coupled Stokes (ω_s) and anti-Stokes (ω_{as}) fields. The two fields are symmetric to the axis of $\boldsymbol{E}_1$ with the same angle of 0.26°, satisfying the phase matching conditions $\boldsymbol{k}_s$=2$\boldsymbol{k}_1$−$\boldsymbol{k}_{as}$ and $\boldsymbol{k}_{as}$=2$\boldsymbol{k}_1$−$\boldsymbol{k}_s$. Then probe field $\boldsymbol{E}_p$ (ω_p, $\boldsymbol{k}_p$, G_p, 400 μW) interjects with $\boldsymbol{E}_1$ inside the Rb cell of also 0.26°, which could

be treated as injected into Stokes or anti-Stokes field, depending on $\omega_p=\omega_s$ or $\omega_p=\omega_{as}$; another field is then called conjugate. Thus the intensities of both Stokes and anti-Stokes fields are amplified and the PA-FWM is formed.

If $\boldsymbol{E}_p$ is injected into Stokes field, the output photons of Stokes and anti-Stokes are

$$N_s=\langle \hat{a}^+_{out}\hat{a}_{out}\rangle=\frac{1}{2}[\cos(2t\sqrt{AB}\sin\frac{\varphi_1+\varphi_2}{2})+\cosh(2t\sqrt{AB}\cos\frac{\varphi_1+\varphi_2}{2})]|\alpha|^2=G|\alpha|^2, \quad (1)$$

$$N_{as}=\langle \hat{b}^+_{out}\hat{b}_{out}\rangle=\frac{1}{2}\frac{B}{A}[\cosh(2t\sqrt{AB}\cos\frac{\varphi_1+\varphi_2}{2})-\cos(2t\sqrt{AB}\sin\frac{\varphi_1+\varphi_2}{2})]|\alpha|^2=(G-1)|\alpha|^2, \quad (2)$$

where $\hat{a}^+(\hat{a})$ is the creation (annihilation) operator that acts on the electromagnetic excitation of the Stokes field, whereas $\hat{b}^+(\hat{b})$ acts on the anti-Stokes field; $|\alpha|^2$ denotes the intensity of injected probe field; A, B, ϕ_1, ϕ_2 are the modulus and phase angles of $\rho^{(3)}_{21(s)}$ and $\rho^{(3)}_{20(as)}$, respectively. Here $\rho^{(3)}_{21(s)}=Ae^{i\varphi_1}$ and $\rho^{(3)}_{20(as)}=Be^{i\varphi_2}$ are the density matrix elements which could be obtained by the Liouville pathways (perturbation chains) $\rho^{(0)}_{11}\xrightarrow{\omega_1}\rho^{(1)}_{21}\xrightarrow{\omega_{as}}\rho^{(2)}_{01}\xrightarrow{\omega_1}\rho^{(3)}_{21(s)}$ and $\rho^{(0)}_{00}\xrightarrow{\omega_1}\rho^{(1)}_{20}\xrightarrow{\omega_s}\rho^{(2)}_{10}\xrightarrow{\omega_1}\rho^{(3)}_{20(as)}$, shown as

$$\rho^{(3)}_{21(s)}=-iG_{as}G_1^2/(d_{21}d_{01}d'_{21}), \quad (3)$$

$$\rho^{(3)}_{21(as)}=-iG_sG_1^2/(d_{20}d_{10}d'_{20}), \quad (4)$$

where $G_{ij}=\mu_{ij}E_{ij}/\hbar$ ($j=s$ and as) is the Rabi frequency, $\Gamma_{ij}=(\Gamma_i+\Gamma_j)/2$ is the decoherence rate between $|i\rangle$ and $|j\rangle$; Δ_i is the detuning between the resonant transition frequency Ω_i and the laser frequency ω_i of $\boldsymbol{E}_i$, denoted as $\Delta_i=\Omega_i-\omega_i$. And we define $d_{10}=\Gamma_{10}+i(\Delta_1-\Delta_s)$, $d_{01}=\Gamma_{01}+i(\Delta'_1-\Delta_{as})$, $d_{21}=\Gamma_{21}+i\Delta'_1$, $d_{20}=\Gamma_{20}+i\Delta_1$, $d'_{20}=\Gamma_{20}+i(\Delta_1+\Delta'_1-\Delta_s)$, $d'_{21}=\Gamma_{21}+i(\Delta_1+\Delta'_1-\Delta_{as})$. $\rho^{(3)}_{21(s)}$ causes the gain in Stokes field at the off-resonant window $\Delta_1-\Delta_s=0$, while the probe transmission signal of $\rho^{(1)}_{20}$ causes EIA at the same window, where $\rho^{(1)}_{20}=iG_1/(d''_{20}+G_s^2/d'_{01})$, and

$d'_{01} = \Gamma_{01} + i(\Delta_1 - \Delta_s)$, $d''_{20} = \Gamma_{20} + i\Delta'_1$. The corresponding perturbation chain is $\rho_{00}^{(0)} \xrightarrow{\omega_1} \rho_{20}^{(1)}$ of $\boldsymbol{E}_p$. Then the intensity of PA-FWM Stokes field is expressed as $I_s \propto (I_0 - \mathrm{Im}(\rho_{20}^{(1)}) + |\rho_{21(s)}^{(3)}|^2)$. While $I_{as} \propto (I_0 + |\rho_{20(as)}^{(3)}|^2)$ for anti-Stokes intensity, $\rho_{20(as)}^{(3)}$ causes gain at $\Delta'_1 - \Delta_{as} = 0$. All the signals appear when $\Delta_p = \Delta_s$, where $\Delta_p = \Omega_{20} - \omega_p$ is the detuning of probe field.

Similarly, if $\boldsymbol{E}_p$ is injected into anti-Stokes field, Eqs (1 and 2) should be swapped. Then the intensity of PA-FWM anti-Stokes field is $I_{as} \propto (I_0 - \mathrm{Im}(\rho_{21}^{(1)}) + |\rho_{20(as)}^{(3)}|^2)$, where $\rho_{21}^{(1)} = iG_1 / (d''_{21} + G_{as}{}^2 / d'_{10})$ and $d'_{10} = \Gamma_{10} + i(\Delta'_1 - \Delta_{as})$, $d''_{21} = \Gamma_{21} + i\Delta_1$. The perturbation is $\rho_{01}^{(0)} \xrightarrow{\omega_1} \rho_{21}^{(1)}$. Then the gain and EIA appear at $\Delta'_1 - \Delta_{as} = 0$, while the gain of $I_s \propto (I_0 + |\rho_{21(s)}^{(3)}|^2)$ appears at $\Delta_1 - \Delta_s = 0$. All the signals appear when $\Delta_p = \Delta_{as}$.

After that, we add $\boldsymbol{E}_2$ (ω_2, $\boldsymbol{k}_2$, G_2) at the opposite direction of $\boldsymbol{E}_1$, and $\boldsymbol{E}_2$ is treated as injected into Stokes field. $\boldsymbol{E}_2$ acts as a dressing field of 30 mW power, 776 nm, detuned from $5P_{3/2}$ ($|2\rangle$) to $5D_{5/2}$ ($|3\rangle$). The presence of $\boldsymbol{E}_2$ will lead to another EIA of probe transmission signal at the window of $\Delta_1 + \Delta_2 = 0$, then $\rho_{20}^{(1)}$ should be modified as $\rho_{20}^{\prime(1)} = iG_1 / (d''_{20} + G_s{}^2 / d'_{01} + G_2{}^2 / d_{30})$ and $d_{30} = \Gamma_{30} + i(\Delta_1 + \Delta_2)$, the perturbation chain is still $\rho_{00}^{(0)} \xrightarrow{\omega_1} \rho_{20}^{(1)}$. If the EIA window of $\rho_{20}^{\prime(1)}$ $(\Delta_1 + \Delta_2 = 0)$ overlaps with the PA-FWM gain window of $\rho_{21(s)}^{(3)}$ $(\Delta_1 - \Delta_s = 0)$, the four-level inverted Y-type six-wave mixing (SWM) atomic configuration is formed, which is co-existing with FWM.

Here we introduce the fifth-order density matrix elements by the perturbation chain $\rho_{00} \xrightarrow{\omega_1} \rho_{20} \xrightarrow{\omega_2} \rho_{30} \xrightarrow{-\omega_2} \rho_{20} \xrightarrow{-\omega_s} \rho_{10} \xrightarrow{\omega_1'} \rho_{20(as)}^{(5)}$ (anti-

Stokes field) $\rho_{11}\xrightarrow{\omega_1'}\rho_{21}\xrightarrow{\omega_2}\rho_{31}\xrightarrow{-\omega_2}\rho_{21}\xrightarrow{-\omega_{as}}\rho_{01}\xrightarrow{\omega_1}\rho_{21(s)}^{(5)}$ and (Stokes field), where

$$\rho_{21(s)}^{(5)}=iG_1^2G_2^2G_{as}/(d_{21}^2d_{31}d_{01}d_{21}') \tag{7}$$

$$\rho_{20(as)}^{(5)}=iG_1^2G_2^2G_s/(d_{20}^2d_{30}d_{10}d_{20}'), \tag{8}$$

$d_{30}=\Gamma_{30}+i(\Delta_1+\Delta_2)$ and $d_{31}=\Gamma_{31}+i(\Delta_1'+\Delta_2)$.

The PA-SWM phase matching conditions should be $\boldsymbol{k}_s=2\boldsymbol{k}_1-\boldsymbol{k}_{as}+\boldsymbol{k}_2-\boldsymbol{k}_2$ and $\boldsymbol{k}_{as}=2\boldsymbol{k}_1-\boldsymbol{k}_s-\boldsymbol{k}_2+\boldsymbol{k}_2$. Then the intensity of Stokes field should be $I_s\propto(I_0-\mathrm{Im}(\rho_{20}'^{(1)})+|\rho_{21(s)}^{(3)}+\rho_{21(s)}^{(5)}|^2)$, while the intensity for anti-Stokes (conjugate) field is $I_{as}\propto(I_0+|\rho_{20(as)}^{(3)}+\rho_{20(as)}^{(5)}|^2)$. And the co-existing of PA-FWM and PA-SWM could be shown as dressed-FWM, i.e. the FWM signal dressed by $\boldsymbol{E}_2$. When the dressing effect of $\boldsymbol{E}_2$ is involved, Eqs (3 and 4) can be modified as

$$\rho_{21(s)}'^{(3)}=-iG_{as}G_1^2/\left(d_{21}+G_2^2/d_{31}\right)d_{01}\left(d_{21}'+G_2^2/d_{31}'\right), \tag{9}$$

$$\rho_{21(as)}'^{(3)}=-iG_sG_1^2/\left(d_{20}+G_2^2/d_{30}\right)d_{10}\left(d_{20}'+G_2^2/d_{30}'\right), \tag{10}$$

where $d_{31}'=\Gamma_{31}+i(\Delta_1'-\Delta_{as}+\Delta_1+\Delta_2)$, $d_{30}'=\Gamma_{30}+i(\Delta_1-\Delta_s+\Delta_1'+\Delta_2)$. And it could be proved that $\rho_{21(s)}'^{(3)}=\rho_{21(s)}^{(3)}+\rho_{21(s)}^{(5)}$ and $\rho_{20(as)}'^{(3)}=\rho_{20(as)}^{(3)}+\rho_{20(as)}^{(5)}$.

Experimentally, we scan the probe detuning Δ_p over 14.0×2π GHz when Δ_1 is around −1.1×2π GHz, then the signals in both probe and conjugate fields are shown on spectrum. There are four positions that trigger multi-wave mixing conditions: Δ_p=6.4×2π GHz, 1.9×2π GHz, −1.1×2π GHz, −4.1×2π GHz, corresponding to $\omega_p-\omega_1$=−7.5×2π GHz, =−3.0×2π GHz, 0 GHz, =3.0×2π GHz, respectively. Here $\omega_p-\omega_1<0$ means probe beam is injected into Stokes field, while $\omega_p-\omega_1>0$ shows probe injected into anti-Stokes field, $\omega_p-\omega_1=0$ GHz is where probe and pump field are

resonant. Specifically, at $\omega_p-\omega_1=-7.5\times2\pi$ GHz, the probe signal is ^{87}Rb, F=2→F' transition and the conjugate signal is ^{87}Rb, F=1→F'; at $\omega_p-\omega_1=-3.0\times2\pi$ GHz, the probe field is ^{85}Rb, F=3→F' transition and the conjugate ^{85}Rb, F=2→F'; at $\omega_p-\omega_1=3.0\times2\pi$ GHz, the probe and conjugate fields stem from ^{85}Rb, F=2→F' and ^{85}Rb, F=3→F' transitions, respectively. It could be observed that the two points of ^{85}Rb ($\omega_p-\omega_1=-3.0\times2\pi$ and $3.0\times2\pi$ GHz) are symmetric to $\omega_p-\omega_1=0$, and the $6\times2\pi$ GHz difference between the two points is the frequency gap between Stokes and anti-Stokes fields. The Stokes and anti-Stokes fields are strongly correlated both in frequency and spatial domains.

Then to focus on the signal characteristics. The conjugate signals only show PA-FWM gain peaks, which are greater in the right than left. As for probe field, there exists wide dip among the signals due to Raman absorption, and the signals at the three points perform the combination of EIA dip and gain peak: at $\omega_p-\omega_1=-7.5\times2\pi$ GHz, there is only dip visually, since the EIA effect is much stronger than gain; at $\omega_p-\omega_1=-3\times2\pi$ GHz, there is co-existence of dip and peak; whereas at $\omega_p-\omega_1=3\times2\pi$ GHz the gain overtakes EIA dip then only peak prevails.

In this paper we investigate mostly on the ^{85}Rb signals at $\omega_p-\omega_1=-3\times2\pi$ and $3\times2\pi$ GHz. Generally, the Stokes and anti-Stokes signals raise exactly at the same Δ_p in each case, while sometimes they have a deviation, which stems from the phase mismatch in frequency. We treat Stokes and anti-Stokes fields as beams with bandwidths, and we can denote ϖ_s, ϖ_{as} as the central angular frequencies of Stokes and anti-Stokes signals in the perfect phase match condition, then the realistic angular frequencies are obtained

as $\omega_s=\varpi_s+\delta$ and $\omega_{as}=\varpi_{as}-\delta$, where δ denotes the phase-mismatch in frequency. Thus Eqs (9 and 10) could be expressed as

$$\rho_{21(s)}^{\prime(3)}(\delta)=\frac{-iG_{as}G_1^{\ 2}e^{i\Delta kr}}{[d_{21}+\frac{|G_2|^2}{i(\Delta_1'+\Delta_2)+\Gamma_{31}}](\Gamma_{01}-i\delta)[\Gamma_{21}+i(\Delta_1-\delta)+\frac{|G_2|^2}{i(\Delta_1+\Delta_2-\delta)+\Gamma_{31}}]}, \quad (11)$$

$$\rho_{20(as)}^{\prime(3)}(\delta)=\frac{-iG_sG_1^{\ 2}e^{i\Delta kr}}{[d_{20}+\frac{|G_2|^2}{i(\Delta_1+\Delta_2)+\Gamma_{30}}][\Gamma_{10}+i\delta][\Gamma_{20}+i(\delta+\Delta_1')+\frac{|G_2|^2}{i(\delta+\Delta_1'+\Delta_2)+\Gamma_{30}}]}. \quad (12)$$

Here we introduce $\boldsymbol{e}^{\Delta \boldsymbol{k}\bullet \boldsymbol{r}}$, where the vector $\Delta\boldsymbol{k}$ is the phase mismatch in space. The equation$\Delta\boldsymbol{k}=2\boldsymbol{k}_1-\boldsymbol{k}_{as}-\boldsymbol{k}_s+\boldsymbol{k}_2-\boldsymbol{k}_2=2(\omega_1/c)\boldsymbol{k}_{10}-((\varpi_{as}-\delta)/v_{as})\boldsymbol{k}_{as0}-((\varpi_s+\delta)/v_s)\boldsymbol{k}_{s0}=\delta((1/v_{as})\boldsymbol{k}_{as0}-(1/v_s)\boldsymbol{k}_{s0})$ shows the relationship between $\Delta\boldsymbol{k}$ and δ, where v_s, v_{as} are the group velocities of Stokes and anti-Stokes signals, and $\boldsymbol{k}_{10}$, $\boldsymbol{k}_{s0}$, $\boldsymbol{k}_{as0}$ are the unit vectors of pump, Stokes and anti-Stokes beams, respectively. Since phase mismatch indicates the multi-mode of signals, the relationship of δ and $\Delta\boldsymbol{k}$ demonstrates the concord of spatial and frequency (temporal) multi-mode. With the mismatch, the outcome of Stokes and anti-Stokes will be modified as

$$N_s=\langle\hat{a}_{out}^+\hat{a}_{out}\rangle=[C^2+(\Delta kS)^2/(2K)^2]|\alpha|^2+(\kappa S/K)^2, \quad (13)$$

$$N_{as}=\langle\hat{b}_{out}^+\hat{b}_{out}\rangle=(\kappa S/K)^2(1+|\alpha|^2), \quad (14)$$

where z is the interaction length of waves, κ is the real part of the nonlinear coefficient for Stokes/anti-Stokes signal ($\kappa=\kappa_s=\kappa_{as}$), C=cosh(Kz), S=sinh(Kz), $K=\sqrt{\kappa^2-(\Delta k/2)^2}$ if $\kappa^2>(\Delta k/2)^2$, and C=cosh(Kz), S=sinh(Kz), $K=\sqrt{(\Delta k/2)^2-\kappa^2}$ if $\kappa^2\leq(\Delta k/2)^2$.

And we use two photo wave packet function:

$$\Psi_{s-as}(\tau)=C_0\int e^{-i(\omega_s\tau_s+\omega_{as}\tau_{as})}\Phi(\Delta kL)\chi_{\omega_{as}}^{(3)}(\delta)d\delta$$

$$= C_0 \int e^{-i(\varpi_s \tau_s + \varpi_{as} \tau_{as})} \mathrm{sinc}[\frac{\Delta k L}{2}] e^{-i\Delta k L/2} \chi^{(3)}_{\omega_{as}}(\delta) e^{i\delta\tau} d\delta \qquad (15)$$

to illustrate the correlation between Stokes and anti-Stokes photons, where nonlinear coefficient $\chi^{(3)}_{\omega_{as}}(\delta)$ associates with δ, and longitudinal detuning function $\Phi(\Delta k\mathrm{L})$ associates with $\Delta \boldsymbol{k}$. As the existence of phase mismatch, there will appear spatial multi-mode.
